# Supplementary material for: Chemical combinations potentiate human pluripotent stem cell-derived 3D pancreatic progenitor clusters toward functional β cells
Source: Nat Commun. 2021 Jun 7;12:3330. doi: 10.1038/s41467-021-23525-x (PMC8184986; doi:10.1038/s41467-021-23525-x)
Supplement: Supplementary file 1 — Supplementary Information [file 41467_2021_23525_MOESM1_ESM.pdf]

Chemical combinations potentiate human pluripotent stem cell-derived 3D pancreatic progenitor clusters toward functional β cells

Haisong Liu et al.

File name: Supplementary Information

Description: Supplementary Figures, Supplementary Tables and Supplementary Methods.

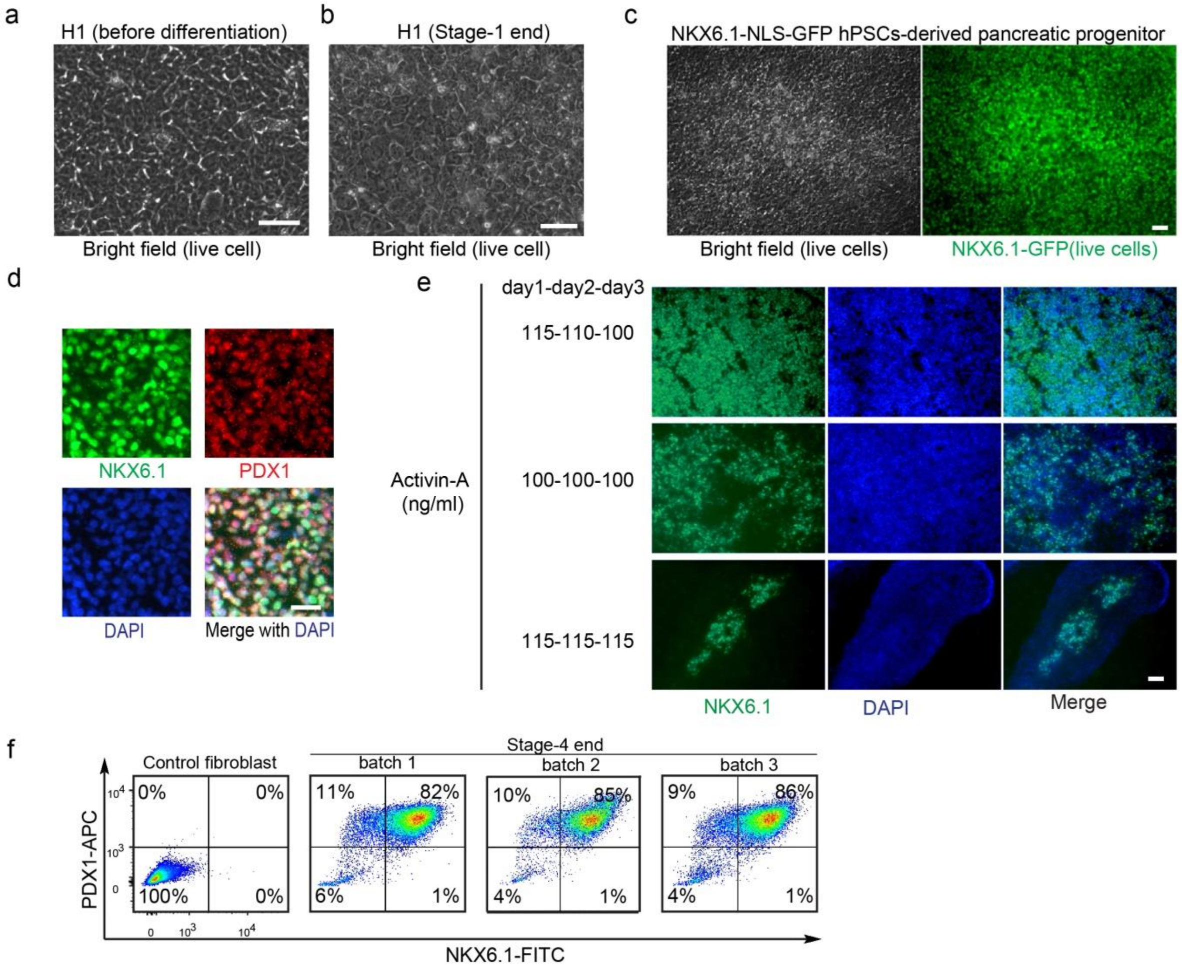

Supplementary Fig. 1. Additional data on the efficiently generating pancreatic progenitor (PP) 3D-clusters, Related to Fig. 1. (a) Morphology of hPSCs right before differentiation. (b) Morphology of hPSCs-derived definitive endoderm cells (at the end of stage 1). (c) Fluorescent image of live pancreatic progenitors generated from NKX6.1-NLS-GFP hPSCs at the end of stage 4. (d) Immunostaining of PDX1 and NKX6.1 of the stage-4 culture cells (e) Immunostaining of NKX6.1 of the stage-4 culture cells after different Activin-A treatment strategies at stage 1. (f) Flow cytometry analysis of stage-4 culture cells from three different batches (n=3). Nuclear DAPI staining is shown in blue. Scale bar, 50 μM for a, b and d; 100 μm for the others. Images of a-e are representative of 3 independent experiments, respectively. Source data are provided as a source data file.

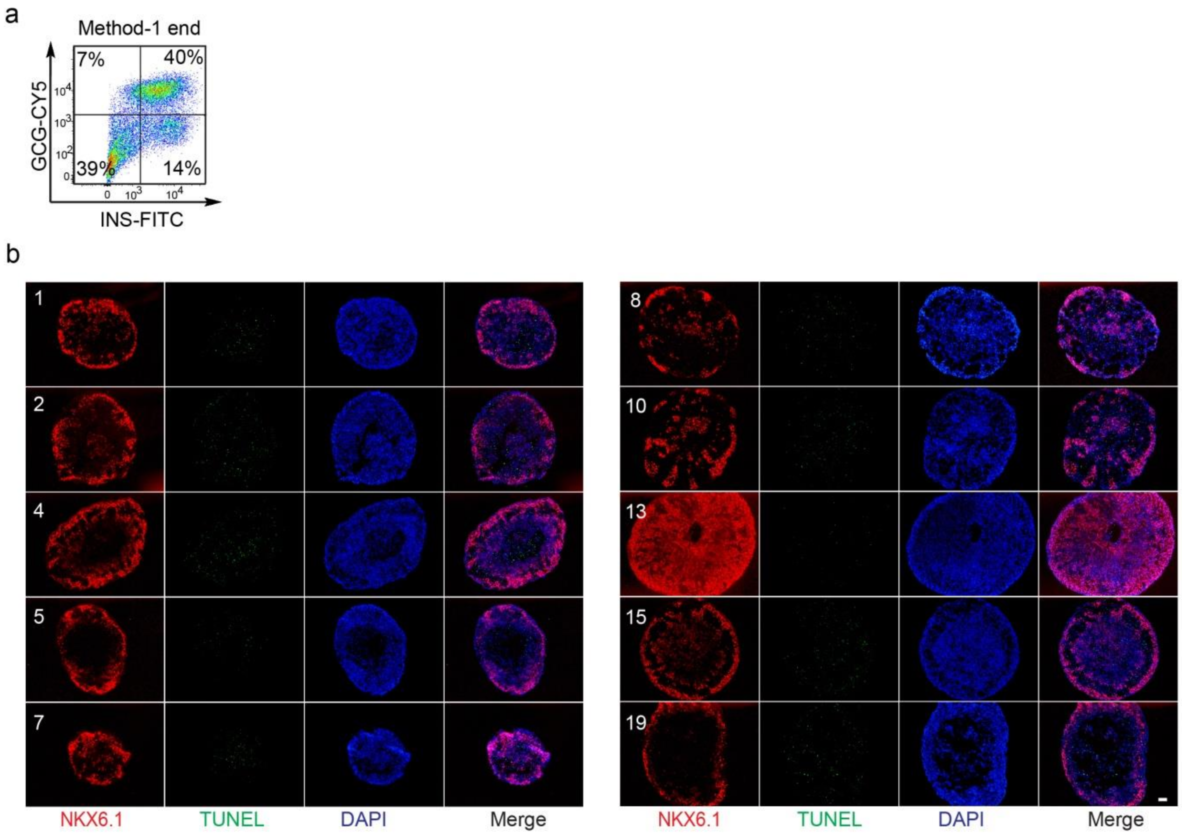

Supplementary Fig. 2. Additional data on the enhancement of the tendency of β cell derivation from pancreatic progenitor (PP) 3D-clusters, related to Fig.2. (a) Flow cytometry analysis of end-stage differentiation cells generated by using Method 1 (n=3). (b) TUNEL analysis of PP-3D clusters after treated by different conditions. Nuclear DAPI staining is shown in blue. Scale bar, 50 µm. Images of b are representative of 3 independent experiments. Source data are provided as a source data file.

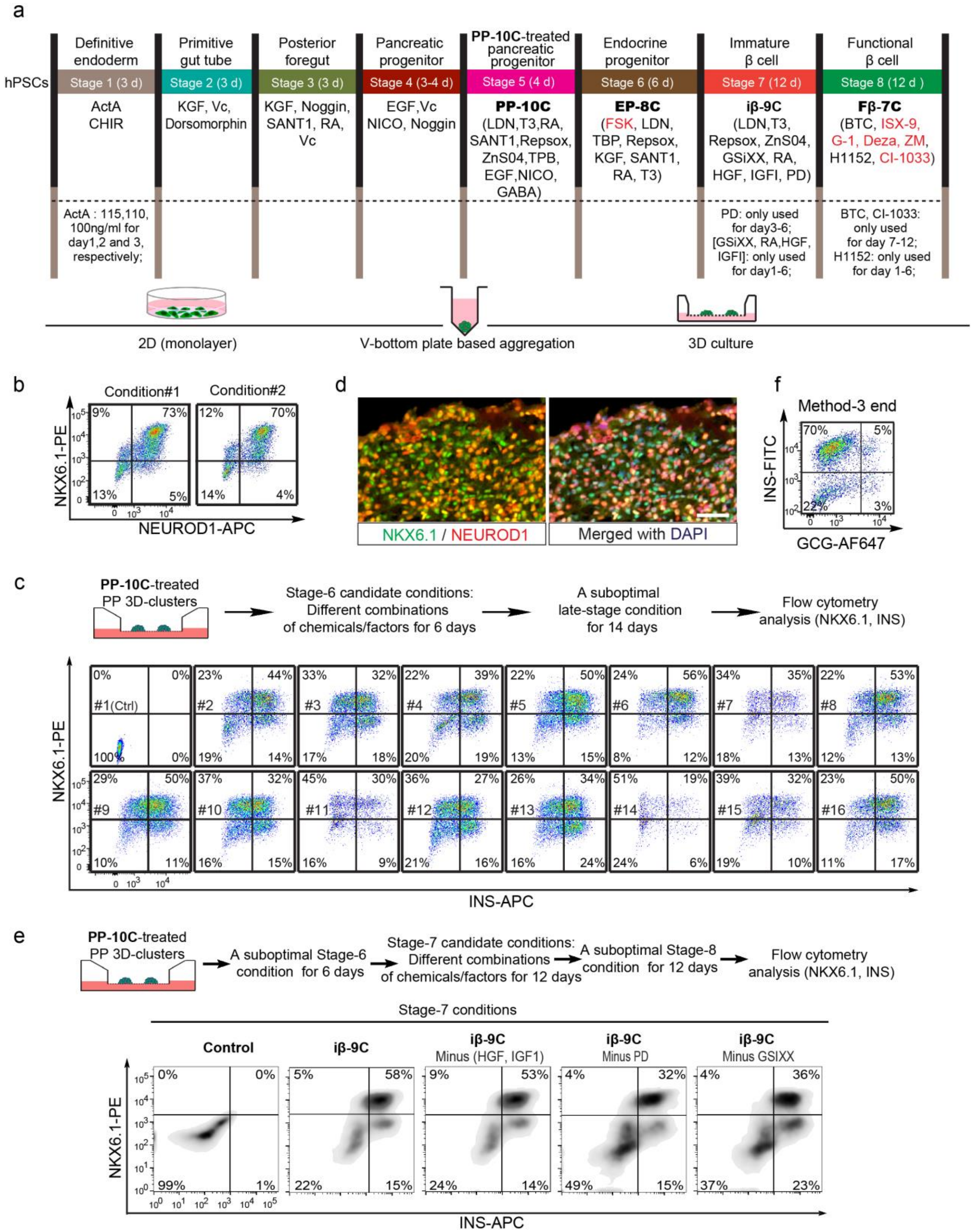

Supplementary Fig. 3. Additional data on the systematic screening for the differentiation of pancreatic progenitors into β cells, Related to Fig. 3. (a) Schematic graph of the entire protocol

established in this study. Chemicals/factors highlighted in red have not been reported in literatures for inducing hPSCs into β cells. (b) PP-10C-treated PPs were incubated with Condition #1 (Cocktail-A)), and Condtion#2 (Cocktail-A+PP2 (pyrrolo-pyrimidine Src family kinase inhibitor, 5 μM)), for 4 days, followed by cell cytometry analysis for the expression of NKX6.1 and NEUROD1. Cocktail-A = $ZnSO4$ + Heparin + LDN (0.1 μM) + T3 (1 μM) + Repsox (10 μM, high concentration) + GXISS (100 nM) + SANT1 (0.25 μM) + RA (0.05 μM) + FSK (10 μM). (c) To obtain a better condition for stage 6 (endocrine progenitor stage), PP-10C-treated PP 3D-clusters were treated with different combinations of chemicals/factors for 6 days, followed by a suboptimal later-stage condition treatment for 14 days. Cells were then analyzed for expression of NKX6.1 and INS using cell cytometry, showing that condition #6 (containing 8 chemicals/factors, termed as EP-8C) generated the most NKX6.1/INS+ cells. (For detailed experimental design, see supplementary method) (n=3). (d) Representative images of NEUROD1 and NKX6.1 immunofluorescence for H1 hES-derived cell cultures at the end of stage 6 after treatment of #6 condition (8 chemicals/factors, EP-8C) at stage 6. (e) Cell cytometry analysis of stage-8 cells showed adding iβ-9C at stage 7 efficiently induced (NKX6.1+/INS+) β cells from earlier stages, while subtracting some factors from iβ-9C at stage 7 reduced the final efficiency (n=3). (f) Cell cytometry analysis of H1 hES-derived stage-8 cells by using Method 3 (n=3). Nuclear DAPI staining is shown in blue. Scale bar, 100 μm. Images of d are representative of 3 independent experiments.

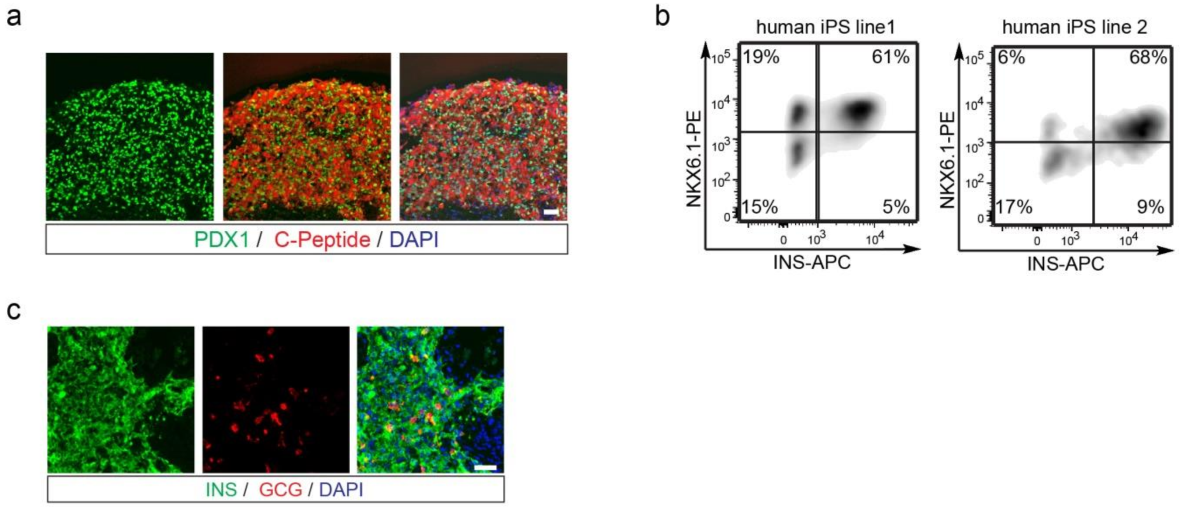

Supplementary Fig. 4. Additional data on the function of the hESCs-derived β cells, Related to Fig. 3. (a) Representative images of PDX1 and C-peptide immunofluorescence in stage-8 cells. (b) Cell cytometry analysis of stage-8 cells from two human iPSC lines. The NKX6.1+/INS+ cell population represents β cells. (c) Representative images of GCG and INS immunofluorescence in a 20-week engraftment derived from stage-8 cells (in normal NSG mice). Scale bar, 100 µm. Source data are provided as a source data file. Images of a and c are representative of 3 independent experiments, respectively.

Supplementary Table 1. Different concentrations of Activin-A used at stage 1 affect the production of NKX6.1+/PDX1+ pancreatic progenitor at the end of stage 4

Activin-A (ng/ml)<br>day1-day2-day3	PDX1+ cells	NKX6.1+ cells	PDX1+/NKX6.+ cells
Constant low-dose of Activin-A<br>(100-100-100)	~50%-80%	~15%-50%	~15%-50%
Decreasing gradient dose of Activin-A<br>(115-110-100)	~93%	~80%	~80%
Constant high-dose of Activin-A<br>(115-115-115)	~90%	~20%-50%	~20%-50%

Supplementary Table 2. A custom library of growth factors and compounds modulating most of the known development and differentiation-related signaling pathways.

Signaling pathway/target	Function (inhibitor/Activator)	Chemical/factor name (concentration tested)
WNT	Inhibitor	XAV939 (0.1-5 μM)
WNT	Inhibitor	wnt-c59 (0.01, 2 μM)
WNT	Activator	wnt3a (2,20 ng/ml)
WNT	Inhibitor	IWR1-1-endo (2.5 μM)
WNT	Inhibitor	IWP2 (0.1-2 μM)
WNT	Activator	CHIR99021 (1,3 μM)
Vitamin	Others	VC (0.25 mM)
Vitamin	Others	Nicotinamide (10 mM)
VEGF	Activator	VEGF (50 ng/ml)
TGFBeta	Activator	TGFBeta3 (1-5 ng/ul)
TGFBeta	Activator	TGFBeta1 (1-5 ng/ul)
TGFBeta	Inhibitor	SB431542 (2 μM)
TGFBeta	Inhibitor	Repsox (1, 2,10 μM)
TGFBeta	Activator	human ActinA (2,10,50 ng/ml)
TGFBeta	Inhibitor	A83-01 (0.5 μM)
TGF (BMP), AMPK	Inhibitor	Dorsomorphin (0.75 μM)
TGF (BMP)	Inhibitor	Noggin (200 ng/ml)
TGF (BMP)	Inhibitor	LDN193189 (0.1 μM)
TGF (BMP)	Activator	human BMP4 (20 ng/ml)
Ribosomal S6 kinase (RSK)	Inhibitor	BRD7389
RHO/ROCK	Inhibitor	Y27632 (10 μM)
RHO/ROCK	Inhibitor	H1152 (5 μM)
RHO/ROCK	Inhibitor	Fasudil (3 μM)
RA (retinoic acid)	Activator	RA (0.01, 1 μM)
RA (retinoic acid)	Inhibitor	LE135 (1 μM)
RA (retinoic acid)	Inhibitor	BMS195614 (1-2 μM)
RA (retinoic acid)	Activator	AM580 (0.05 μM)
RA (retinoic acid)	Inhibitor	AGN193109 (1 μM)
Pluripotency related	others	Minocycline hydrochloride (MiH) (2 μM)
Pluripotency related	others	(S)-(+)-dimethindene maleate (DiM) (2 μM)
PLK4	Inhibitor	Centrinone B (30 nM)
PKC	Activator	TPB (0.2 μM)
PI3K	Inhibitor	Ly294002 (2,20 μM)
PI3K	Activator	Human LIF (10 ng/ml)
PI3K	Activator	Human IGF2 (50 ng/ml)
PI3K	Activator	Human IGF1 (50 ng/ml)

PI3K	Activator	hIGF-II (50 ng/ml)
PDGF	Inhibitor	SU16F (2 μM)
PDGF	Inhibitor	JNJ10198409 (0.1 μM)
PDGF	Activator	human PDGFAB (20 ng/ml)
P70	Inhibitor	LY2584702 (2 μM)
P38,Pluripotency related	Inhibitor	SB202190 (10-20 μM)
P38, MAPK	Inhibitor	SB203580 (20 μM)
Others	Others	Zinc sulfate heptahydrate
Others	Others	Heparin (10 ug/ml)
Others	Others	GABA (1 mM)
Others	Others	dexamethasone
NOTCH-SRC	Inhibitor	PP2 (5 μM)
NOTCH	Activator	JAG-1 (100 ng/ml)
NOTCH	Inhibitor	GSiXX (0.1 μM)
Notch	others	DLl4 (250 ng/ml)
Notch	others	DLl1 (250 ng/ml)
Notch	Inhibitor	DAPT (1 μM)
mTOR	Inhibitor	rapamycin (5 nM)
Monoamine oxidase	Inhibitor	Tranylcypromine (Parnate) (5 μM)
MEK	Inhibitor	PD0325901 (1μM)
Lysophosphatidic acid (LPA)	Activator	LPA (1-2 μM)
JNK, Protein synthesis	others	Anisomycin (5 μM)
JNK	Inhibitor	SP600125 (10-20 μM)
Hypoxia	others	CoCl2 (100-200 μM)
Hormone	Activator	T3 (0.1, 1 μM)
HGF	Activator	HGF (50 ng/ml)
Hedgehog/Smoothened	Inhibitor	sant1 (0.25 μM)
Hedgehog/Smoothened	Activator	Hh-Ag1.5 (0.2 μM)
Hedgehog/Smoothened	Inhibitor	GDC0449 (0.1-2 μM)
GPER	Activator	G-1 (1 μM)
GLP1	Activator	Exendin-4 (50 ng/ml)
Gene activator	NeuroD1 inducer	ISX-9 (10 μM)
Gene activator	PDX1 inducer	BRD7552 (5 μM)
GDNF	Activator	hGDNF (50 ng/ml)
G9a/GLP	Inhibitor	BIX-01294 (1 μM)
FGF	Inhibitor	SU5402 (2 μM)
FGF	Inhibitor	PD173074 (0.1μM)
FGF	Activator	human bFGF (1,5,20 ng/ml)
FGF	Activator	hFgf9 (20 ng/ml)
FGF	Activator	hFGF4 (50 ng/ml)
FGF	Activator	hFGF10 (50 ng/ml)
FGF	Activator	hFGF1 (50 ng/ml)

FGF	Activator	FGF7 (KGF, 5-20 ng/ml)
ER-beta	Activator	WAY-200070 (0.1, 1μM)
ER-beta	Activator	AC-186 (0.5μM)
ER-alpha	Activator	PPT (0.1μM)
ER-alpha	Inhibitor	MPP dihydrochloride (2μM)
Epigenetics	Inhibitor	VPA (0.5 mM)
Epigenetics	Inhibitor	TSA(0.02μM)
Epigenetics	Inhibitor	RG108 (0.1-10μM)
Epigenetics	Inhibitor	NaB (0.2 mM)
Epigenetics	Inhibitor	DEZA; Deazaneplanocin A (1 μM)
Epigenetics	others	5-Azacytidine (AZT, 5μ M)
EGFR and ErbB2	Inhibitor	CI-1033 (1 μM)
EGF related	Activator	Betacellulin (20 ng/ml)
EGF	Activator	hEGF (10,50,100 ng/ml)
EGF	Inhibitor	Erlotinib (10 μM)
DOT1L	Inhibitor	SGC 0946 (2. 5μM)
DOT1L	Inhibitor	EPZ004777 (5 μM)
cAMP, PKA	Activator	8-Br-cAMP (0.1-0.3 mM)
cAMP	Activator	Forskolin (10 μM)
cAMP	Activator	dbcAMP (0.1-1 mM)
Calcium signaling	others	LONO (1 μM)
Calcium signaling	Activator	BayK8644 (2 μM)
AXL,RSK	Inhibitor	R428 (2 μM)
Autophagy	others	SMER28 (50 μM)
Aurora kinase	Inhibitor	ZM447439 (ZM, 0.5, 2.5 μM)
Aurora kinase	Inhibitor	AMG-900 (1 μM)
ATPase	Inhibitor	Blebbistatin (1-10 μM)
Anti-diabetes related	others	Pioglitazone HCl (10 μM)

Supplementary Table 3. Generation of β cells from multiple hPSC lines in different tests.

Cell line	Efficiency of β cell generations in different tests
H1 hESC	78%; 82%; 67%; 75%; 74%
H1 hESC-NKX6.1-GFP	75%; 64%; 77%
hiPSC1	61%; 57%
hiPSC2	68%; 60%; 63%
hiPSC3	63%

Supplementary Method. Other supplementary information.

1. Related to Figure 1J: Experimental design for Figure 1J: To identify the optimal condition for the stage 5, the cells from the stage 4 were dissociated and aggregated (day1) and cultured (day2,3,4) on air-liquid surface using the 21 different conditions as listed in this table. The clusters were then fixed and sectioned for staining. Note: all the factors listed were used for the entire 4 days unless specifically indicated.

Conditions= V4b-mdium+RA(0.05 µM)+SANT1(0.25 µM)+LND(0.1 µM)+T3(1 µM)+ ZnSon4+heparin(10mg/ml)+TPB(0.1 µM)+Y27632(used for only day 1(aggregation day))+ factors listed in give condition					
1	GABA(1mM)				
2	GABA(1mM)	EGF(50ng/ml)	KGF(20ng/ml)		
3					
4	GABA(1mM)	EGF(10 ng/ml)	KGF(20ng/ml)		repsox(10 µM), only for day2,3,4
5	GABA(1mM)		KGF(20ng/ml)		repsox(10 µM), only for day2,3,4
6	GABA(1mM)				repsox(10 µM), only for day2,3,4
7		EGF(100 ng/ml)	NiCO(10mM)	DBZ(1 µM)	repsox(10 µM), only for day2,3,4
8	GABA(1mM)	EGF(100 ng/ml)	NiCO(10mM)	DBZ(1 µM)	repsox(10 µM), only for day2,3,4
9	GABA(1mM)				repsox(10 µM), only for day2,3,4
10					repsox(10 µM), only for day2,3,4
11	GABA(1mM)		KGF(5ng/ml)		repsox(10 µM), only for day2,3,4
12					repsox(10 µM), only for day2,3,4
13	GABA(1mM)	EGF(100 ng/ml)	NiCO(10mM)		repsox(10 µM), only for day2,3,4
14		EGF(10 ng/ml)			repsox(10 µM), only for day2,3,4
15	GABA(1mM)	EGF(10 ng/ml)			repsox(10 µM), only for day2,3,4
16	GABA(1mM)	EGF(10 ng/ml)		DBZ(1 µM)	repsox(10 µM), only for day2,3,4
17	GABA(1mM)	EGF(10 ng/ml)		DBZ(1 µM)	repsox(10 µM), only for day2,3,4
18	GABA(1mM)	EGF(20 ng/ml)	NiCO(10mM)	DBZ(1 µM)	repsox(10 µM), only for day2,3,4
19	GABA(1mM)	EGF(50 ng/ml)	KGF(20ng/ml)		
20		EGF(10 ng/ml)		DBZ(1 µM)	repsox(10 µM), only for day2,3,4
21		EGF(10 ng/ml)			repsox(10 µM), only for day2,3,4

2. Related to Figure S2C: Experimental design for Figure S2C: To identify the optimal condition for the stage 6, the stage 5 clusters were treated with S6-Basic-condtion supplemented with the extra factors as listed for 6 days, followed by treated with later stage medium (made of 4#-medium, supplemented with (gamma secretase inhibitor XX (GXISS, 100nM, only for day1-6)+RA(0.05 µM, only for day1-6)+HGF(50ng/ml, only for day1-6)+IGF1(50 ng/ml, only for day1-6) +FGF inhibitor PD173074 (0.1 µM, only for day3-6)) +BTC(10 ng/ml). 4#-medium=V4b-medium+ ZnS04(10mM)+ Heparin(10mg/ml) + LDN(100 nM)+T3(1 µM)+Repsox(10 µM)). In the end the clusters were dissociated into single cells for cell cytometry analysis for the expression of INS and NKX6.1.

**S6-Basic-condtion=DMEM+B27+LDN(0.5 µM) +TBP(30 nM)+ KGF(25 ng/ml)**						
ID						
**#2**	SANT1(0.25 µM)	RA(0.05 µM)	FSK(10 µM)		Rep(1 µM)	
**#3**	SANT1(0.25 µM)	RA(0.05 µM)		T3(1 µM)		
**#4**	SANT1(0.25 µM)	RA(0.05 µM)	FSK(10 µM)	T3(1 µM)	Rep(1 µM)	BTC(10ng/ml)
**#5**	SANT1(0.25 µM)	RA(0.05 µM)	FSK(30 µM)	T3(1 µM)		
**#6**	SANT1(0.25 µM)	RA(0.05 µM)	FSK(10 µM)	T3(1 µM)	Rep(1 µM)	
**#7**	SANT1(0.25 µM)		FSK(10 µM)	T3(1 µM)	Rep(1 µM)	
**#8**	SANT1(0.25 µM)	RA(0.05 µM)	FSK(20 µM)	T3(1 µM)		
**#9**	SANT1(0.25 µM)	RA(0.05 µM)	FSK(10 µM)	T3(1 µM)	Rep(1 µM)	extra KGF(25ng/ml)
**#10**	SANT1(0.25 µM)	RA(0.05 µM)	FSK(10 µM)	T3(1 µM)	Rep(1 µM)	EGF(10ng/ml)
**#11**		RA(0.05 µM)	FSK(10 µM)	T3(1 µM)	Rep(1 µM)	
**#12**	SANT1(0.25 µM)	RA(0.05 µM)	FSK(10 µM)	T3(1 µM)	Rep(1 µM)	EGF(50ng/ml)
**#13**	SANT1(0.25 µM)	RA(0.05 µM)	FSK(10 µM)	T3(1 µM)	Rep(20 µM)	
**#14**	SANT1(0.25 µM)			T3(1 µM)	Rep(1 µM)	
**#15**	SANT1(0.25 µM)		FSK(10 µM)	T3(1 µM)		
**#16**	SANT1(0.25 µM)	RA(0.05 µM)	FSK(10 µM)	T3(1 µM)	Rep(10 µM)	

3. An example of cell cytometry gating strategy. The preliminary gates of the starting population: The cell population was plotted by FSC-A/FSC-W and SSC-A/SSC-W, and gates were drawn to remove cell with high SSC-W or FSC-W value (these are doublets or aggregates) (See figure below). Typically the resulting cell group was the major single cell population with most fragmented or clustered cells being excluded. To define the boundary between positive and negative, we performed staining on cells negative for the target protein or using antibody controls (either secondary antibody only or isotopes of primary antibody). In the example below, the upper panel showed the gating of undifferentiated hESCs, which was used as a negative cell control for staining of PDX1-APC and NKX6.1-PE. And the lower panel showed the sample example (hiPSC line 1-derived stage-4 cells) stained with PDX1-APC and NKX6.1-PE antibodies.

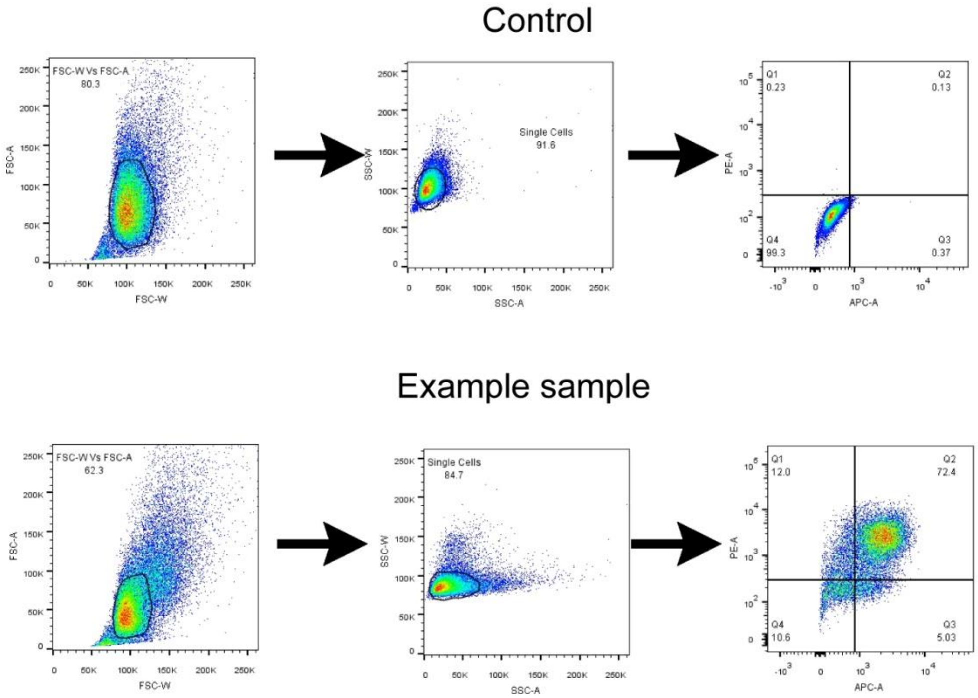

4. Related to Supplementary Figure 1E. Determine the gradient condition of Activin-A at stage 1. At the beginning, we tested Activin-A at 100 ng/ml for 3 days on hPSCs with cell density around 70-80% confluence (a confluence used in several published studies) and found that it could efficiently induce hPSCs into definitive endoderm (>90%, based on the expression of markers FOXA2 and SOX17). However, with such a cell density, we found that it could not generate high percentage of pancreatic progenitors at stage 4. Through further analysis, we discovered that NKX6.1 expression at stage 4 mainly showed up in areas with high cell density. In addition, the lower overall density of the cell culture at stage 1, the less NKX6.1 expression was induced at stage 4 (despite the PDX1 expression was not greatly affected). Therefore, we increased the starting hES cell density to about 90% confluence. However, in such a high cell density we frequently observed a certain amount of undifferentiated hPSCs (~10%-25%) at the end of stage 1, when 100 ng/ml Activin-A was applied constantly for three days at stage 1. Thus, to identify the optimal condition of Activin-A, we examined different concentrations of Activin-A for stage 1, including 80, 90, 95, 100, 105, 110, 115, 120, 125, 130 and 140 ng/ml, and finally the condition of 115 ng/ml worked as the best for the day1 in our hand. Therefore, we decided to use gradient Activin A in our final protocol, which were 115ng/ml (day1), 110ng/ml (day2) and 100ng/ml (day3) as described in Supplementary Fig.1E that either constant high or low dose of Activin-A negatively impacted the NKX6.1 expression. In sum, one possible reason for the use of slightly higher Activin A than most published protocols is that higher starting hPSCs cell density can generate higher NKX6.1 expression at Stage 4, but also requires a higher dose of Activin-A to initiate the differentiation.
