# Supplementary material for: Chemical combinations potentiate human pluripotent stem cell-derived 3D pancreatic progenitor clusters toward functional β cells
Source: Nat Commun. 2021 Jun 7;12:3330. doi: 10.1038/s41467-021-23525-x (PMC8184986; doi:10.1038/s41467-021-23525-x)
Supplement: Supplementary file 3 — Reporting Summary [file 41467_2021_23525_MOESM3_ESM.pdf]

nature research

Corresponding author(s): Juan Carlos Izpisua Belmonte

Last updated by author(s): Apr 22, 2021

# Reporting Summary

Nature Research wishes to improve the reproducibility of the work that we publish. This form provides structure for consistency and transparency in reporting. For further information on Nature Research policies, seeAuthors & Referees and theEditorial Policy Checklist .

## Statistics

For all statistical analyses, confirm that the following items are present in the figure legend, table legend, main text, or Methods section.

| n/a | Confirmed | |
|---|---|---|
| ☐ | ☒ | The exact sample size ($n$) for each experimental group/condition, given as a discrete number and unit of measurement |
| ☐ | ☒ | A statement on whether measurements were taken from distinct samples or whether the same sample was measured repeatedly |
| ☐ | ☒ | The statistical test(s) used AND whether they are one- or two-sided<br>*Only common tests should be described solely by name; describe more complex techniques in the Methods section.* |
| ☐ | ☒ | A description of all covariates tested |
| ☐ | ☒ | A description of any assumptions or corrections, such as tests of normality and adjustment for multiple comparisons |
| ☐ | ☒ | A full description of the statistical parameters including central tendency (e.g. means) or other basic estimates (e.g. regression coefficient) AND variation (e.g. standard deviation) or associated estimates of uncertainty (e.g. confidence intervals) |
| ☐ | ☒ | For null hypothesis testing, the test statistic (e.g. $F$, $t$, $r$) with confidence intervals, effect sizes, degrees of freedom and $P$ value noted<br>*Give P values as exact values whenever suitable.* |
| ☒ | ☐ | For Bayesian analysis, information on the choice of priors and Markov chain Monte Carlo settings |
| ☒ | ☐ | For hierarchical and complex designs, identification of the appropriate level for tests and full reporting of outcomes |
| ☒ | ☐ | Estimates of effect sizes (e.g. Cohen's $d$, Pearson's $r$), indicating how they were calculated |

*Our web collection on statistics for biologists contains articles on many of the points above.*

## Software and code

Policy information about availability of computer code

| | |
|---|---|
| Data collection | Zen 2011 SP7 FP3(balck) (64 bit) used to perform confocal imaging. |
| Data analysis | We used ImageJ(version 1.52i) for digital image analysis, and Graphpad Prism (verion 6.0, version 7, and version 8) for data plotting and statistical analysis. Flow cytometry data was analyzed by FlowJo (Version X.0.7). |

For manuscripts utilizing custom algorithms or software that are central to the research but not yet described in published literature, software must be made available to editors/reviewers. We strongly encourage code deposition in a community repository (e.g. GitHub). See the Nature Research guidelines for submitting code & software for further information.

## Data

Policy information about availability of data

All manuscripts must include a data availability statement. This statement should provide the following information, where applicable:

- Accession codes, unique identifiers, or web links for publicly available datasets
- A list of figures that have associated raw data
- A description of any restrictions on data availability

The raw data associated with study is available from the corresponding author on reasonable request.

# Field-specific reporting

Please select the one below that is the best fit for your research. If you are not sure, read the appropriate sections before making your selection.

☒ Life sciences ☐ Behavioural & social sciences ☐ Ecological, evolutionary & environmental sciences

# Life sciences study design

All studies must disclose on these points even when the disclosure is negative.

| | |
|---|---|
| Sample size | Sample size was not predetermined. Sample size was described in the figure legends or the method section. For all experiments, sample size was at least n=3 biological replicates. These are shown on the figures with dot plots overlaying bar graphs. |
| Data exclusions | No data were excluded from the analyses. |
| Replication | All experiments were repeated as described in the manuscript. |
| Randomization | For experiments involved with mice, mice were randomly allocated into different experimental groups. For other experiments, samples were randomly allocated into different experimental groups. |
| Blinding | Investigators performing tissue collection and analysis were blind to group allocation. |

# Reporting for specific materials, systems and methods

We require information from authors about some types of materials, experimental systems and methods used in many studies. Here, indicate whether each material, system or method listed is relevant to your study. If you are not sure if a list item applies to your research, read the appropriate section before selecting a response.

## Materials & experimental systems

| n/a | Involved in the study | |
|---|---|---|
| ☐ | ☒ | Antibodies |
| ☐ | ☒ | Eukaryotic cell lines |
| ☒ | ☐ | Palaeontology |
| ☐ | ☒ | Animals and other organisms |
| ☒ | ☐ | Human research participants |
| ☒ | ☐ | Clinical data |

## Methods

| n/a | Involved in the study | |
|---|---|---|
| ☒ | ☐ | ChIP-seq |
| ☐ | ☒ | Flow cytometry |
| ☒ | ☐ | MRI-based neuroimaging |

## Antibodies

| | |
|---|---|
| Antibodies used | NKX6.1 (DSHB, F55A12-c, 300x), PDX1(R&D, AF2419, 300x), INS-APC (Cell signaling, C27C9, 80x), NKX6.1-PE (BD, #563023, 40x), Human C-peptide (Abcam, Ab14181, 100x), Glucagon (cell signaling, 2760S, 400x), Glucagon (Abcam, ab82270, 100x), Insulin (Abcam, ab7842, 100x), GFP (Abcam, ab6673, 300x), NEUROD1 (R&D, AF2746, 200x), NEUROD1-APC (BD, 563000, 50x). Secondary antibodies were: Donkey anti-mouse Alexa Fluor 488 (Jackson Lab, 715-545-151), Donkey anti-Goat Alexa Fluor 488 (Jackson Lab, 705-545-147), Donkey anti-Rabbit Alexa Fluor 488 (Jackson Lab, 711-545-152), Donkey anti- Guinea Pig Alexa Fluor 488 (Jackson Lab, 706-545-148), Donkey anti-mouse Alexa Fluor 550 (Invitrogen, SA5-10167), Donkey anti-rat Alexa Fluor 555 (Abcam, ab150154), Donkey anti-Rabbit Alexa Fluor Cy3 (Jackson Lab, 711-165-152), Donkey anti-rat Alexa Fluor 647 (Abcam, ab150155), Donkey anti-Goat Alexa Fluor 647 (Jackson Lab, 705-605-147), Donkey anti-Guinea Pig Alexa Fluor 647 (Jackson Lab, 706-605-148), Donkey anti-mouse Alexa Fluor 647 (Jackson Lab, 715-605-151), Donkey anti-Guinea Pig FITC (Jackson Lab, 706-095-148), Donkey anti-Mouse Cy5 (Jackson Lab , 715-175-151), Donkey anti-Mouse Alexa Fluor 647 (AF647) (Jackson Lab , 715-605-151), and Donkey anti-Rabbit Alexa Fluor 647 (Jackson Lab, 711-605-152). All secondary were prepared following the manufactory' instruction and used as 300-500x. |
| Validation | Various antibody dilutions were tested including the manufacturer's recommended dilution. The secondary antibody only controls/ isotype controls, negative sample controls (samples that do not express a given gene; mostly hESCs in this case), positive sample controls (samples that express a given gene, mostly pancreatic tissues or cells in this case) were used to evaluate and confirm specificity of the antibodies to the respective epitopes. All the antibodies used in this study have been used and reported in prior studies.<br>NKX6.1 (DSHB, F55A12 or F55A12-c): has been cited by 40 papers such as an immunofluorescence application in human pancreatic islet related sample shown by Augsornworawat, P., et al., Cell Rep, 2020. 32(8): p. 108067.<br>PDX1(R&D, AF2419): has been cited by 59 papers such as an immunofluorescence application in human pancreatic islet related sample shown by Mfopou, J.K., et al., Biosci Rep, 2016. 36(3).<br>INS-APC (Cell signaling, C27C9) : has been cited by 62 papers such as an immunofluorescence application in human related sample shown by Shigemoto-Kuroda, T., et al., Stem Cell Reports, 2017. 8(5): p. 1214-1225.<br>NKX6.1-PE (BD, #563023): has been cited by 3 papers and validated in a flow cytometry application for human related sample by the manufactory as shown in (https://www.bdbiosciences.com/us/applications/research/intracellular-flow/intracellular-antibodies-and-isotype-controls/anti-human-antibodies/pe-mouse-anti-nkx61-r11-560/p/563023).<br>Human C-peptide (Abcam, Ab14181): has been cited by 59 papers such as an immunofluorescence application in human related sample shown by Guo, D., et al., Sci Rep, 2017. 7(1): p. 3156.<br>Glucagon (cell signaling, 2760 or 2760S): has been cited by 44 papers such as an immunocytochemistry application in human related sample shown by Gerlach, K., et al., Cancer Res, 2012. 72(17): p. 4340-50. |

Glucagon (Abcam, ab82270): has been cited by 14 papers such as an immunocytochemistry application in human related sample as shown in Peterson, Q.P., et al., Nat Commun, 2020. 11(1): p. 2241.
Insulin (Abcam, ab7842): has been cited by 282 papers such as an immunofluorescence application in human related sample as shown by Ruiz, L., et al., Cell Death Dis, 2018. 9(6): p. 600.
GFP (Abcam, ab667 ): has been validated in a immunofluorescence application for by the manufactory as shown in (https://www.abcam.com/gfp-antibody-ab6673.html).
NEUROD1 (R&D, AF2746): has been cited by 13 papers such as an immunofluorescence application in human related sample as shown by N Sharon, ., et al., Cell Rep, 2019; 27(8): 2281-2291.e5.
NEUROD1-APC (BD, 563000): has been validated in a flow cytometry application by the manufactory as shown in (https://www.bdbiosciences.com/us/applications/research/intracellular-flow/intracellular-antibodies-and-isotype-controls/anti-human-antibodies/purified-mouse-anti-neurod1-r8-294/p/563000)

## Eukaryotic cell lines

Policy information about cell lines

Cell line source(s): The H1 hESC line was obtained from WiCell Research Institute, Inc. (Madison, WI); NKX6.1-GFP line was generated from H1 in this study; human iPS cell line 1 was provided by SALK Stem Cell Core; human iPS cell line 2 and 3 were generated from our lab.

Authentication: Cell line authentication (Karyotyping) was done for H1 hESC line. No authentication for the other cell lines were done.

Mycoplasma contamination: Mycoplasma contamination were checked regularly to make sure all the cell lines are mycoplasma-free. All cell lines tested negative for mycoplasma contamination at the moment they were used for differentiation.

Commonly misidentified lines (See ICLAC register): No such cell line was used

## Animals and other organisms

Policy information about studies involving animals; ARRIVE guidelines recommended for reporting animal research

Laboratory animals: 6-to-8-week-old male NOD-scid IL2 gamma null (NSG) mice were from the Jackson Laboratory. Mice were housed in 12-hr light/12-hr dark cycle, with temperatures 65-75°F and humidity 40-60%.

Wild animals: This study did not involve wild animals

Field-collected samples: Did not involve samples collected from the field

Ethics oversight: All procedures related to animals were performed in accordance with the ethical guidelines of the Salk Institute for Biological Studies. Animal protocols were reviewed and approved by the Salk Institute Institutional Animal Care and Use Committee (IACUC) before any experiments were performed.

Note that full information on the approval of the study protocol must also be provided in the manuscript.

## Flow Cytometry

### Plots

Confirm that:

- [x] The axis labels state the marker and fluorochrome used (e.g. CD4-FITC).
- [x] The axis scales are clearly visible. Include numbers along axes only for bottom left plot of group (a 'group' is an analysis of identical markers).
- [x] All plots are contour plots with outliers or pseudocolor plots.
- [x] A numerical value for number of cells or percentage (with statistics) is provided.

### Methodology

Sample preparation: Single-cell suspension from cell cultures: Differentiated hESC cultures were rinsed with PBS and then incubated with 0.25% trypsin-EDTA (Life Technologies) at 37°C for 1-3 minutes. The trypsin was neutralized with MEF medium. The dissociated cells were rinsed twice in PBS or DMEM/F12 medium for further analysis. For intracellular antibody staining, single cells were fixed in 200 µL of BD Cytofix/Cytoperm Buffer (BD Biosciences) at 4°C for 20 minutes followed by three washes in BD Perm/Wash Buffer. Fixed cells were incubated in 150 µL of primary antibody buffer at 4°C for 1 hour, followed by 30 minutes in a secondary antibody (if there is a secondary antibody) buffer after being rinsed twice in Perm/Wash Buffer. Stained cells were washed twice in Perm/Wash Buffer prior to analyses.

Instrument: BD FACSCanto RUO and other FACS analyzers

Software: The data was acquired by BD software and analyzed by FlowJo.

| | |
|---|---|
| Cell population abundance | No FACS sorting was performed, only analysis. |
| Gating strategy | Gating strategy and a figure exemplifying the gating strategy was provided in the Supplementary Method. Here are an example of cell cytometry gating strategy. The preliminary gates of the starting population: The cell population was plotted by FSC-A/FSC-W and SSC-A/SSC-W, and gates were drawn to remove cell with high SSC-W or FSC-W value (these are doublets or aggregates) (See figure in Supplementary method). Typically the resulting cell group was the major single cell population with most fragmented or clustered cells being excluded. To define the boundary between positive and negative, we performed staining on cells negative for the target protein or using antibody controls (either secondary antibody only or isotopes of primary antibody). In the example  the upper panel showed the gating of undifferentiated hESCs, which was used as a negative cell control for staining of PDX1-APC and NKX6.1-PE. And the lower panel showed the sample example (hiPSC line 1-derived stage-4 cells) stained with PDX1-APC and NKX6.1-PE antibodies. |

☒ Tick this box to confirm that a figure exemplifying the gating strategy is provided in the Supplementary Information.
